# Rapidly progressive glomerulonephritis, thrombotic microangiopathy and amebic colitis: a challenging case report

**DOI:** 10.1186/1546-0096-12-S1-P291

**Published:** 2014-09-17

**Authors:** Adriana Fonseca, Jose Guilherme Leite, Adriana Azevedo, Eugenia Lacerda, Arnould Kaufman, Mara Rocha, Monica Soares, Maria Lucia Caldas, Leonardo Campos

**Affiliations:** 1 Pediatric Rheumatology, Hospital Federal dos Servidores do Estado, Rio de Janeiro, Brazil; 2 Pediatric Nephrology, Hospital Federal dos Servidores do Estado, Rio de Janeiro, Brazil; 3 Hospital Federal dos Servidores do Estado, Rio de Janeiro, Brazil; 4 Pediatric Immunology, Hospital Federal dos Servidores do Estado, Rio de Janeiro, Brazil; 5 Pathology, Bioneo Lab, Rio de Janeiro, Brazil; 6 Pediatric Rheumatology, IPPMG-UFRJ, Rio de Janeiro, Brazil

## Introduction

Thrombotic microangiopathy (TMA) describes a pathological process of microvascular thrombosis, consumptive thrombocytopenia and microangiopathic haemolytic anaemia, leading to end-organ ischaemia and infarction. TMA is a feature of a number of clinical disorders, most commonly hemolytic uremic syndrome (HUS) and thrombotic thrombocytopaenic purpura. Several molecular mechanisms mediating TMA have been elucidated; however, challenges remain in distinguishing specific causes of TMA in the presence of overlapping clinical features.

## Objectives

We aim to report a case of thrombotic microangiopathy, rapidly progressive glomerulonephritis and amebic colitis.

## Methods

A 12 year old previously healthy boy was admitted with acute renal injury, requiring dialysis. Initial lab exams revealed urea of 213 mg/dL, creatinine 6.0 mg/dL, hematocrit 18%, hemoglobin 7 g/dL, thrombocytopenia, LDH 5900 mg/dL, urine protein-to-creatinine ratio of 4.3, and low C3. Due to evolution with myocardial dysfunction and pericardial tamponade, IV methylprednisolone (MP) pulses were started and he developed massive gastrointestinal bleeding due to amebic colitis, evidenced at colonic biopsy. Plasmapheresis, gammaglobulin, metronidazole and teclosan, were initiated, and after infection control: MP pulses, Rituximab, cyclophosphamide and therapeutic low-molecular weight heparin. Renal biopsy revealed crescentic glomerulonephritis, IgG and C3 on glomeruli, and C1q tubular deposits, but also arteriolar TMA. Despite this, he developed end stage renal disease. Serial autoantibodies tests were negative. ADAMTS13 activity assay was 80%. Immunological investigation performed after immunosuppression showed persistent and marked reduction of serum IgA/IgM/IgG and lymphocyte, even after discontinuation of cyclophosphamide and rituximab. This patient currently has mild thrombocytopenia, leukopenia, lymphopenia, laboratorial signs of hemolysis, and livedo reticularis. Genetic studies for complement-mediated diseases are pending.

## Results

This is a case with severe evolution, characterized by rapidly progressive glomerulonephritis and TMA. The main question is whether this patient has atypical HUS, perhaps associated with amebic dysentery, or an antiphospholipid syndrome. The persistently negative autoantibodies, and the partial response to the adopted treatment, made pSLE less probable.

## Conclusion

The persistent active disease and the extreme difficulty of vascular access for renal replacement therapy make it urgent to perform kidney transplantation. Considering the high risk of disease relapse and subsequent loss of renal graft, it is essential to confirm the diagnosis to decide to use eculizumab (anti-C5 monoclonal antibody) or not, and especially to ensure better post-transplant prognosis.

## Disclosure of interest

None declared.

